# Secondary Iron Overload Due to Amino Acid Chelated Iron Supplementation: A Case Report Involving a Mother and Daughter

**DOI:** 10.7759/cureus.77251

**Published:** 2025-01-10

**Authors:** Naomi Sugimori, Yoh Jinnouchi, Toru Mizoguchi

**Affiliations:** 1 Department of Hematology, Sugimori Clinic, Takaoka, JPN; 2 Orthomolecular Medical Nutrition Therapy, Mizoguchi Clinic, Tokyo, JPN; 3 Department of Epidemiology, Fukushima Medical University School of Medicine, Fukushima, JPN

**Keywords:** amino acid chelated iron, ferrochel, ferrous amino acid chelate, ferrous bisglycinate, ferrous bisglycinate chelate, iron, iron bisglycinate chelate, iron overload, iron supplementation, iron supplements

## Abstract

Amino acid chelated iron (bisglycinate chelate iron, Ferrochel) has been developed and used as a food fortifier to prevent and treat iron deficiency anemia. However, its long-term use has not been described and reports of iron overload are unavailable. We report a case of a mother and daughter who were diagnosed with iron overload based on blood sampling. Serum analysis revealed elevated ferritin levels and increased transferrin saturation (TSAT). MRI of the daughter showed iron accumulation in the liver, spleen, and bone marrow with normal inflammatory response and no findings suggestive of collagen disease or tumor, and she was diagnosed with secondary iron overload due to oral administration of an amino acid chelated iron preparation. After discontinuing over-the-counter medications and approximately two years of phlebotomy, the women showed a trend toward improvement in their hematologic profiles.

Although amino acid chelated iron supplements can be easily administered without side effects such as nausea, ferritin levels should be checked if they are administered for more than a year. Even asymptomatic patients may suffer from iron overload, which can be very dangerous if left untreated. Therefore, amino acid chelated iron should be considered as medicine rather than as a supplement, and immediate action is needed in these cases. This report highlights the importance of careful medication review of over-the-counter amino acid chelated iron supplements.

## Introduction

Artificial iron pills have been developed to solve the global iron shortage and are widely available. However, their long-term use is still a matter of controversy. Amino acid chelated iron (bisglycinate chelate iron, Ferrochel) has been developed and used as a food fortifier to prevent and treat iron deficiency anemia, and it has been associated with minimal gastrointestinal symptoms and other side effects [1]. To date, most reports on the use of amino acid chelated iron have involved short-term clinical trials, with no reports on long-term use of more than a year.

The absorption of amino acid chelated iron is regulated by the amount of stored iron [2], and it has been speculated that iron overload does not occur in these instances; however, iron amino acid chelate preparations, which are thought to be absorbed via an amino acid transporter different from the absorption pathway of nonheme iron and heme iron, may cause iron overload [3]. We report the case of a 45-year-old mother and her 15-year-old daughter who developed iron overload after the intake of amino acid chelated iron preparations for more than a year.

## Case presentation

Case 1

The patient was a 15-year-old female, diagnosed with a developmental disorder at seven years of age, and had a history of taking antipsychotics. She had started taking amino acid chelated iron at 12 years of age on the recommendation of her mother who had read in a book that “iron administration is effective for developmental disorders.” At 13 years of age, she had experienced an onset of nausea, leading her to take the supplement only occasionally, and she had eventually stopped taking it at 15 years of age due to stomatitis and swollen gums. Because her symptoms persisted, her mother took her to the Sugimori Clinic in April 202X and had a blood test, the results of which are presented in Table 1.

**Table 1:** Laboratory test results for Case 1 (15-year-old daughter) Blood tests were performed after four years of amino acid chelated iron consumption. No other abnormal values of AST, ALT, and γGTP were observed. The ferritin level was 2194.1 (ng/mL). After 100 mL of phlebotomy monthly for two years, the ferritin level dropped to 733.5 (ng/mL) ALT: alanine aminotransferase; AST: aspartate aminotransferase; Fe: iron; Hb: hemoglobin; HCT: hematocrit; MCH: mean corpuscular hemoglobin; MCHC: mean corpuscular hemoglobin concentration; MCV: mean corpuscular volume; TIBC: total iron-binding capacity; TSAT: transferrin saturation; γGTP: gamma-glutamyl transpeptidase

Variable	Normal value	Examination findings at the initial visit
		April 202X
Hb (g/dL)	11.5-16.0	14.9
HCT (%)	34.0-47.0	41.2
MCV (fL）	84-100	90.2
MCH (pg)	26-36	32.6
MCHC (g/dL)	32-36	36.2
Fe (μg/dL)	43-172	157
TIBC (μg/dL)	251-398	296
Ferritin (ng/mL)	5.0-177.6	2194.1
TSAT (%)	20-50	53
AST (IU/L)	12-31	28
ALT (IU/L)	8-40	37
γGTP (IU/L)	9-49	26

**Figure 1:**
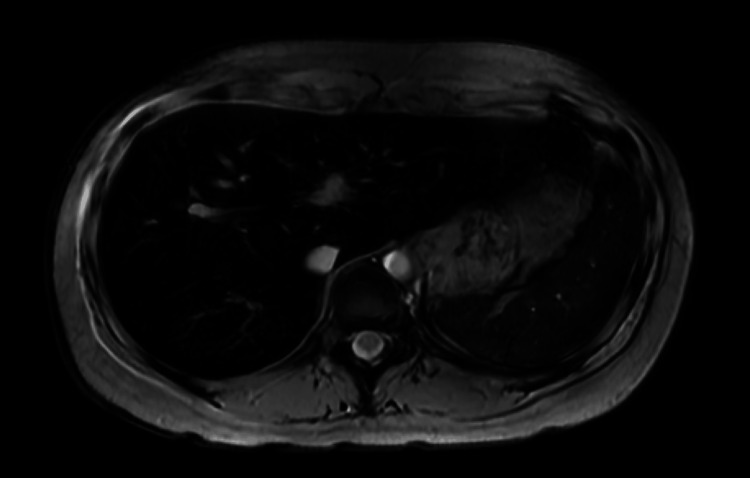
Liver MRI T2*-weighted image: the liver and spleen are relatively more sensitive to magnetic substances such as iron in the tissues than the muscle and bone marrow. The diagnosis reached was iron overload secondary to the oral administration of amino acid chelated iron (bisglycinate chelate iron, Ferrochel) MRI: magnetic resonance imaging

At the initial visit, the serum ferritin level was 2194 ng/mL, with a moderate increase and a transfer saturation (TSAT) of 53%. Liver MRI (Figure 1) showed iron accumulation in the liver, spleen, and bone marrow (radiology imaging at Kouseiren Takaoka Hospital); the inflammatory response [C reactive protein (CRP)] was normal; no findings suggestive of collagen disease or tumor were observed, and secondary iron overload due to oral administration of amino acid chelated iron preparations was diagnosed. Phlebotomy treatment of 100 mL was performed once a month, and the ferritin concentration dropped to 733.5 ng/mL in January 202X+2. Phlebotomy entails the removal of a certain amount of blood from a patient for therapeutic purposes. The nurse at the clinic manually performed the phlebotomy with a syringe, and considering the burden on the patient and the nurse, 100 ml of phlebotomy was given each time.

Case 2

This case involves the mother of Case 1: a 45-year-old woman. She had been diagnosed with hyperlipidemia and uterine fibroids at 25 and 35 years old, respectively. She had started taking 27 mg of the amino acid chelated iron twice daily at the same time as her daughter. The mother had excessive menstruation due to uterine fibroids, and hence she voluntarily took the iron supplement that her daughter was taking. She had not done a gynecologist test for anemia. She had become concerned while visiting the outpatient clinic with her daughter, leading her to visit the Sugimori Clinic in August 202X and undergo a blood test, although she was asymptomatic. Table 2 shows the initial laboratory findings.

**Table 2:** Laboratory test results for Case 2 (45-year-old mother) Blood tests were performed after four years of amino acid chelated iron consumption. No other abnormal AST, ALT, and γGTP values were observed. The ferritin level was 1691.1 (ng/mL). After 100 ml of phlebotomy monthly for two years, the ferritin level dropped to 518.1 (ng/mL) ALT: alanine aminotransferase; AST: aspartate aminotransferase; Fe: iron; Hb: hemoglobin; HCT: hematocrit; MCH: mean corpuscular hemoglobin; MCHC: mean corpuscular hemoglobin concentration; MCV: mean corpuscular volume; TIBC: total iron-binding capacity; TSAT: transferrin saturation; γGTP: gamma-glutamyl transpeptidase

Variable	Normal value	Examination findings at the initial visit
		August 202X
Hb (g/dL)	11.5-16.0	13.9
HCT (%)	34.0-47.0	40.7
MCV (fL)	84-100	90
MCH (pg)	26-36	30.8
MCHC (g/dL)	32-36	34.2
Fe (μg/dL)	43-172	104
TIBC (μg/dL)	251-398	299
Ferritin (ng/mL)	5.0-177.6	1691.1
TSAT (%)	20-50	35
AST (IU/L)	12-31	20
ALT (IU/L)	8-40	34
γGTP (IU/L)	9-49	53

Ferritin level was moderately elevated at 1691 ng/mL and TSAT was 35% in blood sampling at the initial visit. She was diagnosed with secondary iron overload due to intake of amino acid chelated iron preparations. Normally, treatment is carried out with drugs that bind to iron and excrete it from the body; however, due to side effects, the patient had to undergo phlebotomy. We also administered phlebotomy treatment once a month, and in July 202X+1, the ferritin level dropped to 518.1 ng/mL; phlebotomy was discontinued, and the patient was kept under observation.

## Discussion

Iron overdose has been reported to occur even with the consumption of oral iron tablets [4]. Cases of suicides related to nonheme iron overdose [5], ferritin overdose, and chronic diarrhea have been reported [6]. While amino acid-chelated iron overdose has not been reported so far, overdose could occur with long-term use, such as in the cases presented here. Further experimental evaluation is needed to more comprehensively elucidate the mechanism of AA-/peptide-mediated iron absorption by iron glycine chelates [7]. One possible mechanism is that iron glycine chelates are not absorbed through the native physiological iron absorption pathways, such as DMT1 and HCP1. Although orally administered iron is originally shed from the tip of the small intestinal villi and excreted via the stool, iron amino acid chelates accumulate in the crypts of the small intestinal epithelium [3].

## Conclusions

We reported two cases of iron overload in a mother and her daughter who were diagnosed using blood tests and MRI. Based on their medical history, the cause was thought to be the ingestion of an amino acid chelated iron supplement (bisglycinate chelate iron, Ferrochel). Chelated iron supplements carry a risk of iron overload, and ferritin levels should be checked in cases of long-term use. Iron overload can occur even in asymptomatic patients and can be dangerous if left untreated. Chelated iron glycinate should be administered as medicine, not as an iron supplement, and immediate action is needed in these cases.
